# Supplementary material for: Auditory processing ability in Thai native speakers with the Gaps-In-Noise (GIN) test
Source: PeerJ. 2025 Nov 6;13:e20207. doi: 10.7717/peerj.20207 (PMC12598593; doi:10.7717/peerj.20207)
Supplement: Supplemental Information 3 [file peerj-13-20207-s003.docx]

**Data dictionary**

| Variable | Position | Label | Measurement Level |
| --- | --- | --- | --- |
| ID | 1 | <none> | Nominal |
| Ath_bothear | 2 | A.th. both ear (msec) | Scale |
| correct_bothear | 3 | Percent correct both ear (%) | Scale |
| Ath_Rtear | 4 | A.th. right ear (msec) | Scale |
| correct_Rtear | 5 | Percent correct right ear (%) | Scale |
| Ath_Ltear | 6 | A.th. left ear (msec) | Scale |
| correct_Ltear | 7 | Percent correct left ear (%) | Scale |
